# Unicortical self-drilling external fixator pins reduce thermal effects during pin insertion

**DOI:** 10.1007/s00068-017-0887-2

**Published:** 2017-12-14

**Authors:** Markus Greinwald, Patrick A. Varady, Peter Augat

**Affiliations:** 1 Institute of Biomechanics, Trauma Center Murnau, Prof.-Küntscher-Str. 8, 82418 Murnau, Germany; 2 Institute of Biomechanics, Paracelsus Medical University, Salzburg, Austria

**Keywords:** External fixation, Thermographic camera, Unicortical pin, Thermal necrosis, Pin insertion

## Abstract

**Introduction:**

External fixation is associated with the risk of pin loosening and pin infection potentially associated to thermal bone necrosis during pin insertion.

**Objective:**

This study aims to investigate if the use of external fixator systems with unicortical pins reduces the heat production during pin insertion compared to fixators with bicortical pins.

**Methods:**

Porcine bone specimens were employed to determine bone temperatures during insertion of fixator pins. Two thermographic cameras were used for a simultaneous temperature measurement on the bone surface (top view) and a bone cross-section (front view). Self-drilling unicortical and bicortical pins were inserted at different rotational speeds: (30–600) rpm. Maximum and mean temperatures of the emerging bone debris, bone surface and bone cross-section were analyzed.

**Results:**

Maximum temperatures of up to 77 ± 26 °C were measured during pin insertion in the emerging debris and up to 42 ± 2 °C on the bone surface. Temperatures of the emerging debris increased with increasing rotational speeds. Bicortical pin insertion generated significantly higher temperatures at low insertion speed (30 rpm)

**Conclusion:**

The insertion of external fixator pins can generate a considerable amount of heat around the pins, primarily emerging from bone debris and at higher insertion speeds. Our findings suggest that unicortical, self-drilling fixator pins have a decreased risk for thermal damage, both to the surrounding tissue and to the bone itself.

## Introduction

Open fractures with severe soft tissue damage or swelling with risk of compartment syndrome often require a two-staged treatment approach with initial management by external fixation and subsequent treatment with an intramedullary nailing or plating [1, 2]. External fixation is also being employed for bone transport maneuvers to treat large bone defects [3] or to correct malalignments or dysplasias. Transdermal pins required for external fixation constitute a gate for bacteria and are associated with an increased risk of infection, both superficial as well as deep infections into the medullary canal [4, 5]. Thus, it is usually recommended to convert the external fixator to an internal fixation in less than 2 weeks [1, 2].

The most frequent complication in external fixation is infection of the pin tract and pin loosening [6, 7] which is thought to be related to necrosis of the bone and the surrounding tissue [8–10]. Especially for self-drilling pins at high rotational speeds the temperatures occurring on the bone surface can reach levels that are detrimental to biological tissue. Temperatures around 80 °C were measured during the insertion of 5 mm pins without predrilling [11]. New pin-to-bar fixation systems with unicortical pins try to address both intramedullary infection and thermal necrosis. With unicortical pins the medullary canal is not penetrated and the risk of intramedullary infection might be diminished. Moreover, as unicortical pins need not to be drilled through the entire cortex the heat production during insertion is likely to be reduced compared to bicortical pins penetrating both cortices.

This study aims to investigate if the use of fixator systems with unicortical pins reduces the heat production during pin insertion compared to fixators with bicortical pins. We hypothesized that the temperatures occurring for the unicortical system would be lower than those occurring for the bicortical system. Temperature profiles within the bone and on the bone surface were analyzed during pin insertion with different rotational speeds.

## Methods

### Test setup

**Fig. 1:**
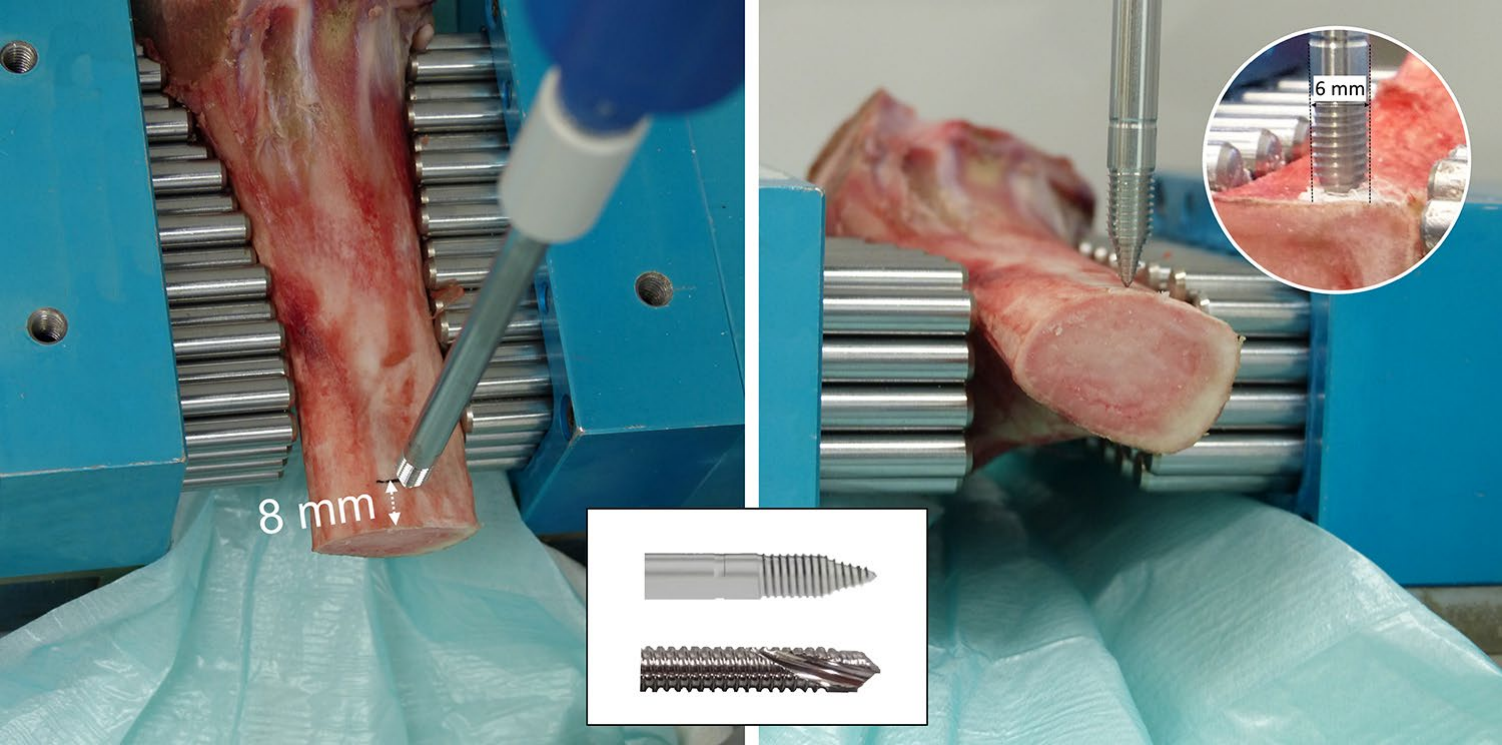
Viewing directions [top (left) and front view (right)] of the two thermal imagers. Pin positioning in 8 mm distance from the surface of cutting surface. Comparison of uni- (upper middle) and bicortical (lower middle) screw tips. Inserted unicortical screw (upper right corner)

Self-drilling pins from two different fixator systems were compared in this study: 12 unicortical pins (UNYCO, ORTHOFIX Srl, Bussolengo, IT, REF 93507, pin diameter: 6 mm) and 12 bicortical (Hoffmann II pins Stryker Corp, Kalamazoo, US-MI, REF 5018-6-180, pin diameter: 5 mm). The unicortical screws were inserted with the provided torque limiter according to the surgical technique. By inserting the pins into the cortical bone of the shaft area the torque limiter triggers before the tip is fully countersunk in the bone surface resulting in an effective diameter smaller than 6 mm (Fig. 1). This was compensated by using a smaller pin diameter for the bicortical comparison group.

**Fig. 2:**
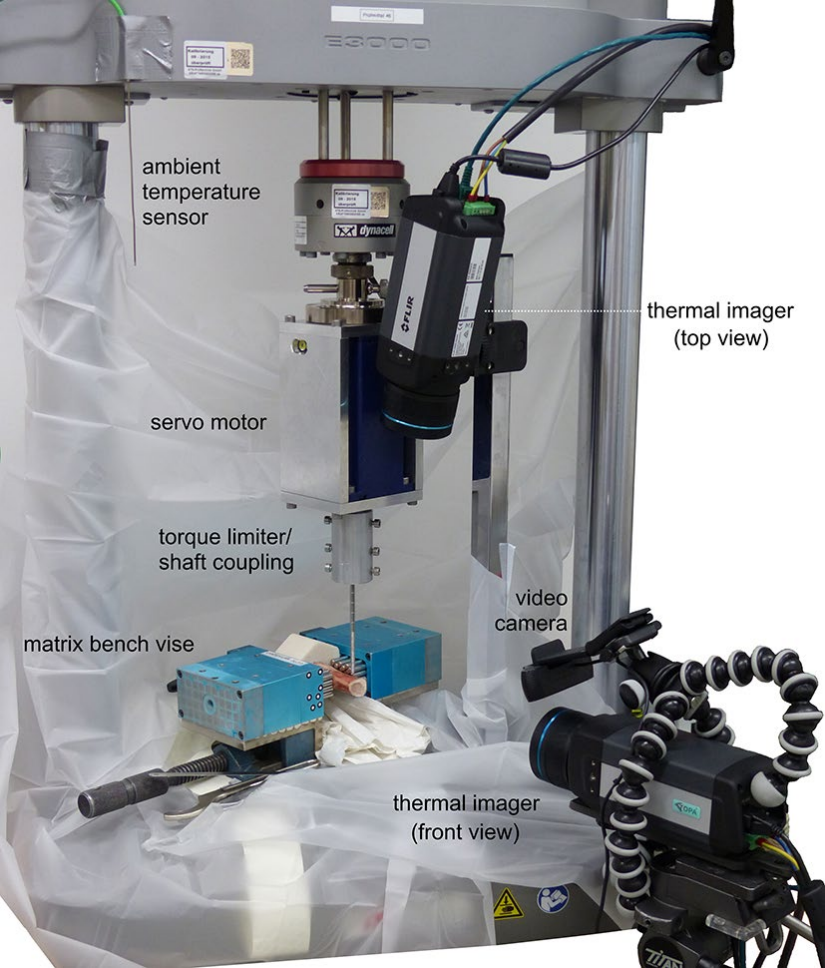
Test setup with two thermal imagers aimed onto the surface and the cross-section of the bone

The pins were inserted into fresh-frozen porcine tibiae (age: 6 months) provided by the local butcher. They were cut in halves to create cross-sectional areas and thawed until they reached room temperature (23 °C). Viewing directions of the cameras were defined as top view for the bone surface and front view for the cross-section of the bone (see Figs. 1, 2). Pin insertion was performed without predrilling in the tibial diaphysis at a distance of 8 mm from the cutting surface (Fig. 1).

The pins were inserted with a servo motor (PD6-N89, Nanotec Electronic GmbH & Co. KG, Feldkirchen, GER) which was mounted on an Instron E3000 material testing machine (Instron Ltd, High Wycombe, UK). The bones were fixed to the machine frame with a bench vise (MATRIX GmbH, Ostfildern, GER). The heat propagation was continuously monitored by two thermographic cameras (FLIR A655sc, Flir Systems, Inc., Portland, US-OR) from the start of pin insertion, triggered by the testing machine, until 3 min after complete insertion of each pin. According to the datasheet, the cameras had a resolution of 640 × 480 pixel, a noise equivalent temperature difference (NETD) of < 30 mK and an accuracy of ± 2 °C or ± 2% of reading. Ambient room temperature was kept constant by climate control and was continuously measured by a K101 digital thermometer (Voltcraft, Wollerau, CH).

To minimize off-center movement of the pin and to simulate the pin positioning of the surgeon before drilling, the pin touched the bone surface with a small force of 3 N prior to initiation of the drilling procedure. At 10 N preload the digital position of the testing machine was zeroed and a constant axial load of 100 N was applied. For both groups (unicortical and bicortical) three pin insertions were tested, respectively, for four rotational speeds (30, 225, 450, and 600 rpm), resulting in a total of 24 experiments. After each insertion a new unused pin was used.

**Fig. 3:**
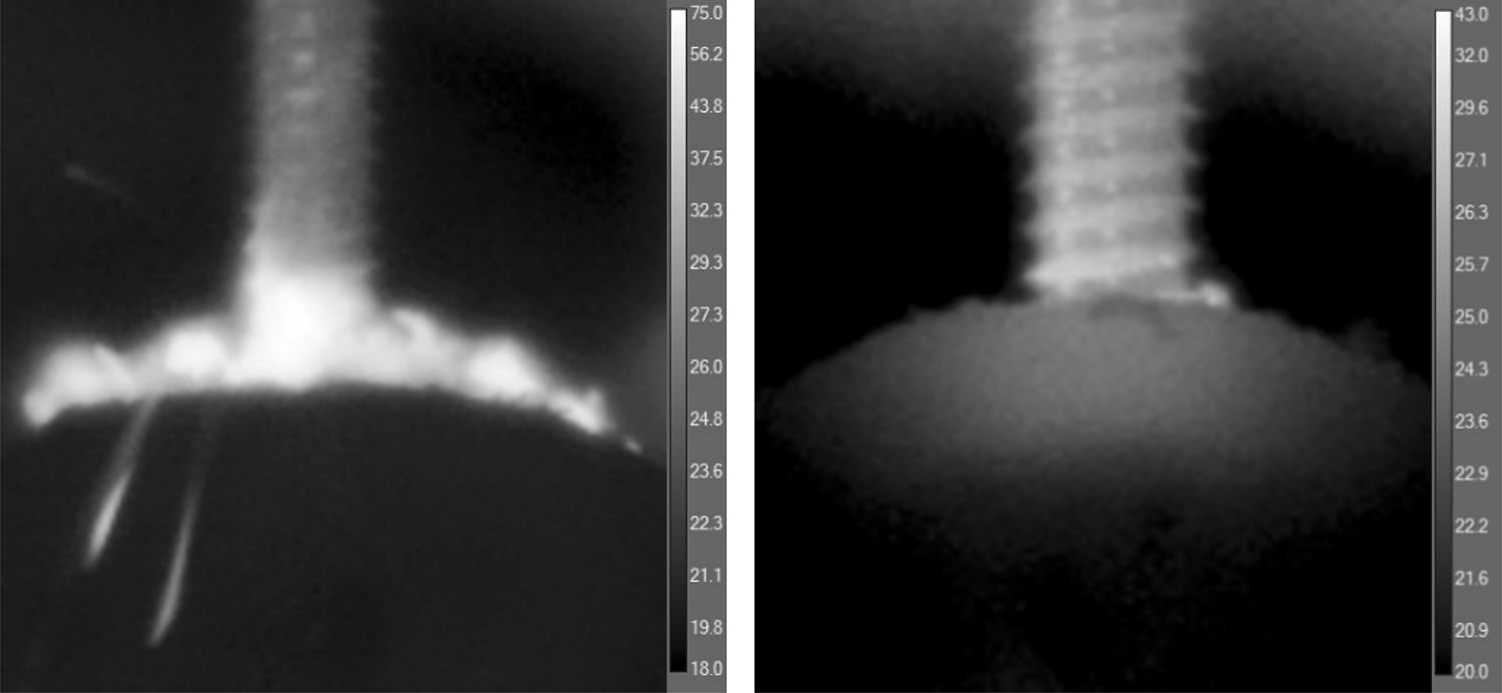
Front view during pin insertion (left) with maximum temperatures of 75° within the bone debris and directly (< 1 s.) after removal of the debris showing the temperature increase in the bone (right)

For the unicortical pins, the pin insertion stopped either with the triggering of the torque limiter provided with the system or at an insertion depth of 15 mm (marked by a circular depression directly on the pin as a maximum insertion depth). For the bicortical pins the pin insertion was stopped when the pin penetrated the opposite cortex. A short burst of compressed air (< 1 s) was applied to the upper cortex directly after complete pin insertion (Fig. 3). The purpose of this was to remove bone debris originating from the drilling to provide an unrestricted view onto the drilling site for the upper infrared camera.

Testing was performed at room temperature (23 °C). Bones were alternated for testing and sawing (to create the new cross-sections) in such a way that they would always cool down again to ambient temperature. 12 halved bone specimens were circled through the testing and sawing process.

### Data analysis

**Fig. 4:**
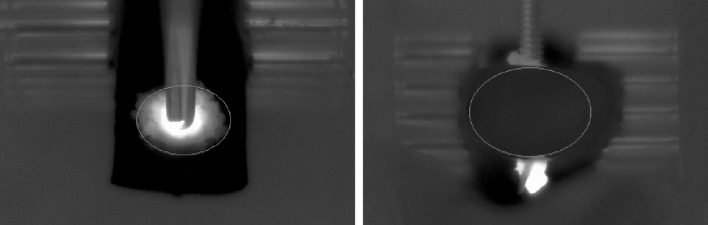
Exemplary regions of interest (ROI) in the thermographic images in top (left) and front view (right)

For both camera views an elliptical mask was fitted on the region of interest representing two surface areas of 179 mm^2^ (top view) and 320 mm^2^ (front view) (Fig. 4) that were prone to temperature changes. In the top view the maximum temperature which occurred during the insertion process was assessed. As the top view always included the pin it was confirmed that the maximum temperature would not occur on the pin by adjusting the ROI placement accordingly.

Furthermore, to visualize the amount of bone affected by the rise in temperature, the top view was further analyzed. After removal of the bone debris with compressed air the area in mm^2^ for each specific temperature within the top view ROI was determined.

Statistical analysis was performed by Student’s *t* test for independent samples comparing the unicortical and bicortical pins for each rotational speed. Level of significance was set to *α* = 0.05. Calculations were performed in Microsoft Excel 2010 (Microsoft Corp, Redmond, US-WA).

## Results

**Fig. 5:**
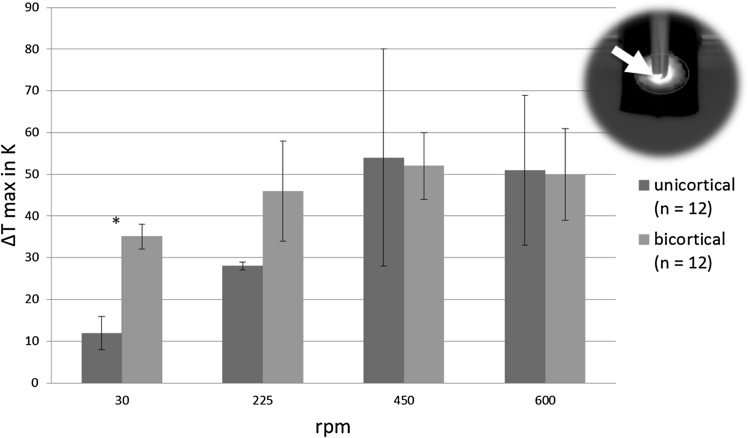
Mean maximum temperature increase of the bone debris ± standard deviations in the top view (*p < 0.05)

**Fig. 6:**
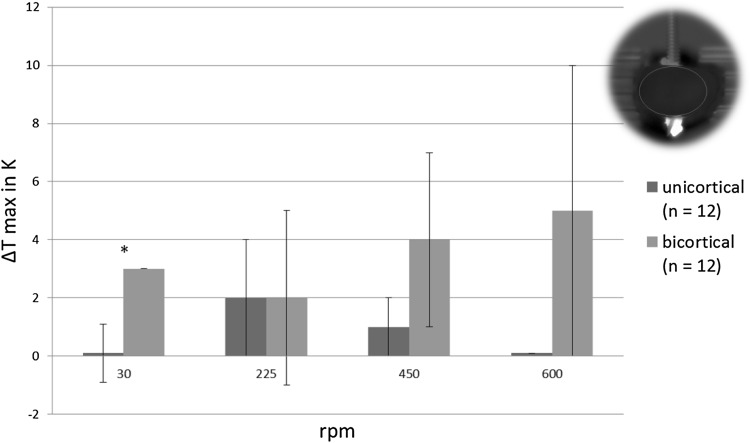
Mean maximum temperature increase of the bone cross-section ± standard deviations in the front view (*p < 0.05)

For both types of pins the maximum temperature in the top view including the bone debris increased with increasing rotational speeds until 450 rpm, but not beyond (Fig. 5). The unicortical pins created significantly smaller increase in temperatures at 30 rpm in both camera perspectives (Figs. 5, 6). In the front view maximum temperatures did not exceed 33 °C, i.e., temperature increase remained below 10 K (Fig. 6).

**Fig. 7:**
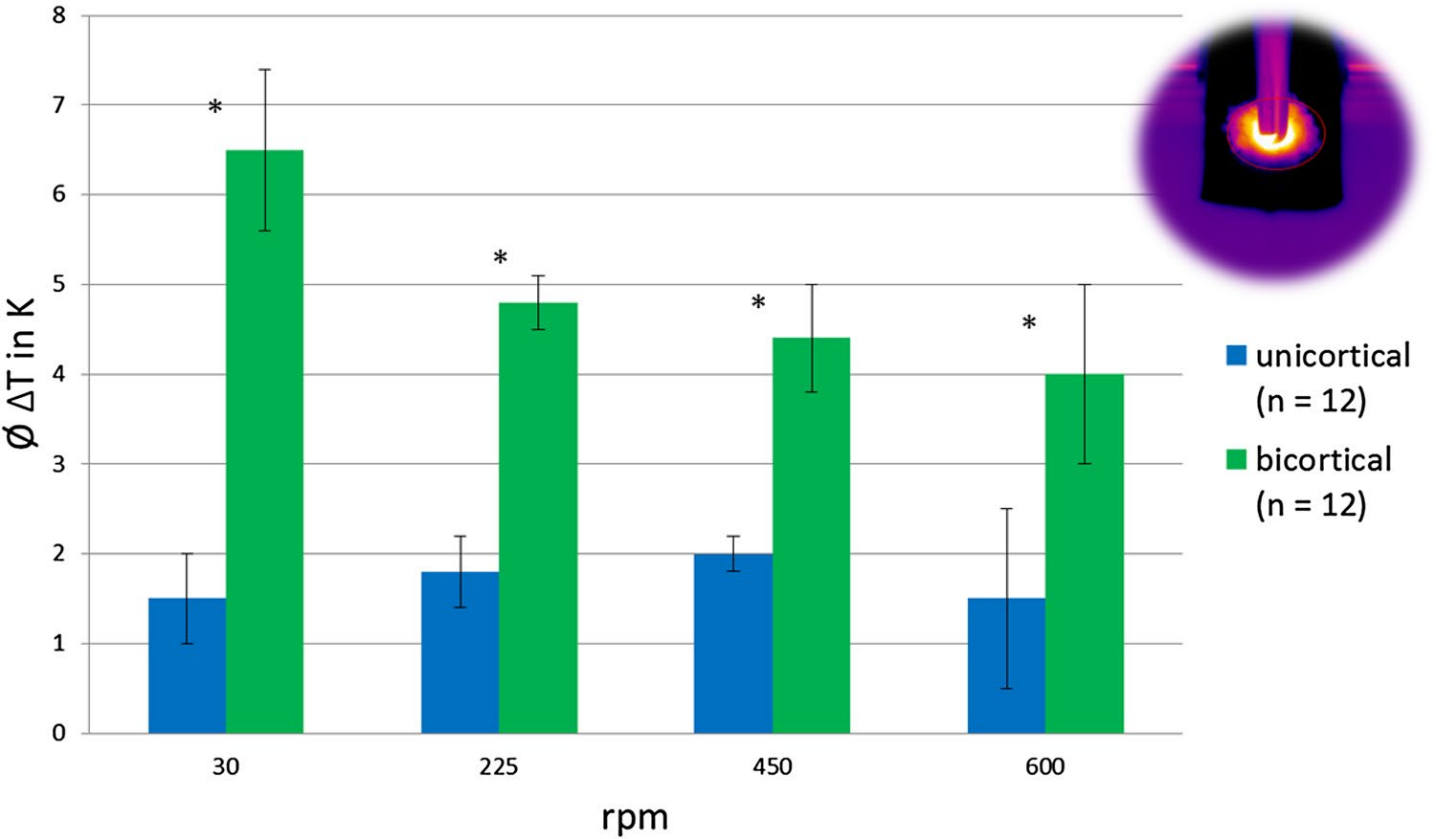
Mean temperature increase of the bone surface ± standard deviations (*p < 0.05)

**Fig. 8:**
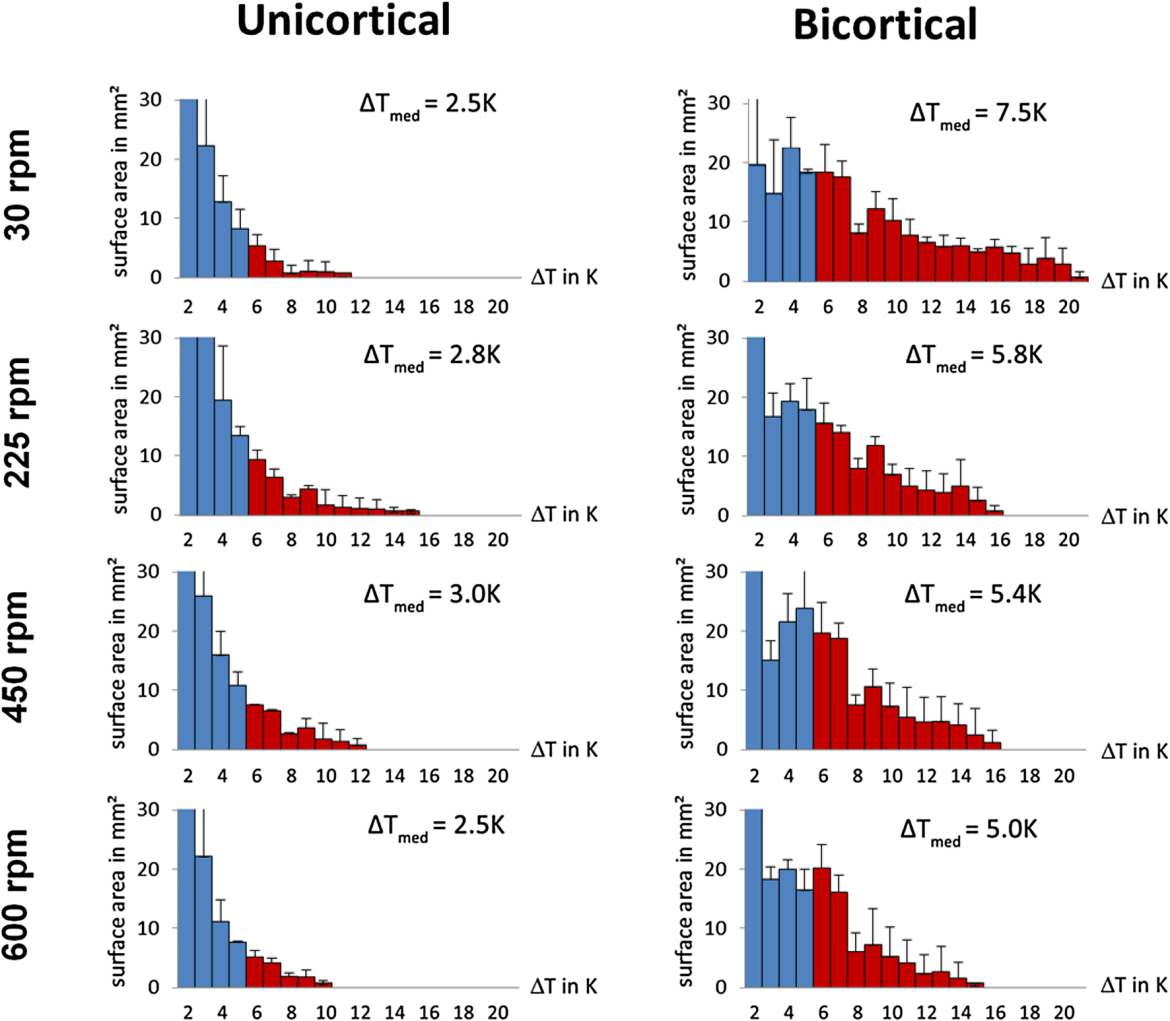
Histogram of temperature increase measured as surface area on the bone surface (top view). Red bars indicate a temperature increase of 6 K and above

The bone surface that showed a temperature increase was considerably larger during bicortical insertion than during unicortical insertion for all rotational speeds (Fig. 7). The temperature increase within the bone was always more pronounced for bicortical pin insertion compared to unicortical pin insertion (Fig. 8). Unicortical pins affected much less bone tissue (Figs. 7, 8).

**Fig. 9:**
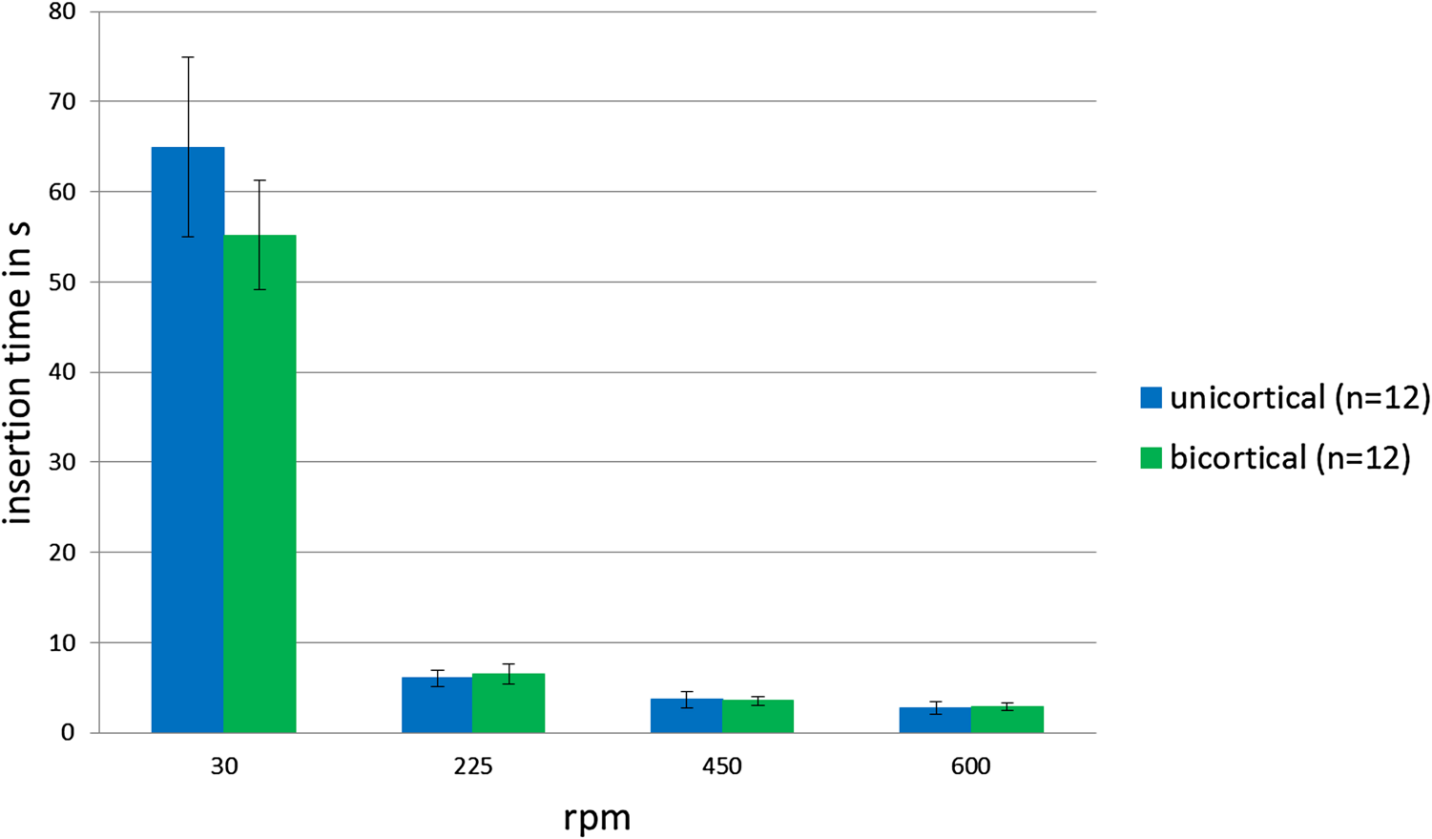
Mean insertion time ± standard deviations (*p < 0.05)

Time of insertion was measured starting at 10 N preload until triggering of the torque limiter (monocortical) or perforation of the second cortex (bicortical). No significant differences were found between the groups within the same rotational speed (Fig. 9).

## Discussion

In this study on heat production of external fixator pins during drilling into bone, we were able to detect differences between unicortical and bicortical pin designs. Insertion of bicortical pins created higher temperatures in the emerging bone debris and on the bone surface compared to insertion of unicortical pins. These differences strongly depended on the speed of drilling. For drilling speeds of 450 rpm and above both types of pins generated temperatures which could potentially induce osteonecrosis or necrosis of the surrounding tissues. According to the literature temperatures over 70 °C could lead to instant bone necrosis [12, 13]. For drilling speeds of 225 rpm and below, the unicortical pins generated significantly lower temperatures compared to bicortical pins resulting in significantly smaller potential thermic tissue damage for the unicortical pins.

The test setup proved to be a feasible method to measure the surface and emerging material temperatures while drilling pins into bone. In our experiments the sample size was limited by the single use specification and therefore the number of pins. Statistical analysis thus was based on only 3 observations per combination of pin type and rotational speed clearly limiting the statistical power of our experimental study.

The thermocameras employed for the measurement of temperature and potential biological damage were ideally suited to monitor the time dependent behavior of the bone temperature during the drilling procedure. Although the stated accuracy of ± 2 °C or ± 2% of reading seems imprecise this value only refers to absolute values of general conditions. Using relative values with constant parameters (materials, emission factor, focus, lightning, etc.) the value should get closer to the NETD of < 30 mK that refers to the resolution of the sensor pixels. The accuracy of our readings should be in between those two values.

Inherent to the detected infrared signal is that the measurement was only based on the temperatures on the bone surface. Using a second camera measuring the temperature in the cross-section perpendicular to the pin direction we could to some extent monitor the heat progression within the bone. The front view measurement heavily depended on the thickness of bone between the drill bore and the bone surface. We were not able to drill closer to the cross-section of the bone as this would have potentially resulted in breakage of the bone bridge between the cutting surface and the drill hole. The high standard deviations of the front view resulted from small variations in pin positioning due to unguided (not pre-drilled) pin placement. Nevertheless these values show reasonable tendencies.

Another limitation of this study is that it was conducted at room temperature and ex vivo. Also using cooling fluids or simply blood from the patients which has not been considered in our test setup might have affected the temperature values. Absolute temperature values will probably differ from in vivo conditions, but despite these limitations, the relative temperature changes and ratio of induced thermal energy observed in this series of experiments are probably transferable to in vivo conditions. The measured temperatures also agree with findings from previous studies [11] in which bone temperatures of up to (77.3 ± 12.7) °C were measured with Synthes self-drilling pins at 400 rpm. Also it has to be stated that this unicortical system requires a higher number of pins (minimum three per fragment, bicortical: two per fragment) to achieve its stability and therefore, the risk of infection is increased accordingly [14]. Also predrilling of the bicortical pins would lead to lower temperature values. Finally, the temperature development will also depend on various design features of the screws, including thread pitch and self-cutting design. Thus, the observed temperature differences between unicortical and bicortical insertion might partly be due to differences in these design features.

Our findings confirm that manual drilling, represented by an insertion speed of 30 rpm, will result in lower temperatures for the emerging bone debris (especially for the unicortical pins). But for bicortical pins this will result in a larger area with high temperatures on the bone surface. This could be attributed to the fact that the hot bone debris cannot be sufficiently evacuated from the pin-hole at low rpm. Therefore it increases the temperature inside the bone. Only for the unicortical pin group at 30 rpm the mean maximum temperatures remained within a physiological temperature range. Rotational speeds greater than 450 rpm show detrimental magnitudes for both types of pins. The influence of tissue damage on biomechanical stability is a topic that would certainly need additional research.

In contrast to the top view, the front view did not show any detrimental temperature ranges. This was most likely due to the thickness of the bone in between the cross-sectional surface and the pin surface. The analysis of the temperature distribution profile was only feasible for the top view, but not for the side view. Despite this limitation, the front views provided information on the temperature distribution and also showed significant differences between the two pin types.

The temperature within the bone after removal of the debris was much lower than the temperature of the debris generated during drilling. The temperature distribution profiles show that bicortical pins will result in larger areas with higher temperature increases compared to unicortical pins, thus much more thermal energy was generated by the bicortical pins. As one might expect the generated thermal energy is (apart from others, e.g., coefficient of friction, pin diameter, rotational speed) a function of the number of rotations required for the pin insertion. Therefore, due to their mechanical principles, bicortical pins need more rotations and thus generate more heat. In general, drills or pins with larger diameter are expected to generate higher temperatures [15]. The 5 mm pin diameter of the comparison group was chosen due to the clinical practice of our consultant surgeons. The unicortical pins which were thicker by 1 mm seem to compensate for this effect with their unicortical insertion technique and possibly their different tip design.

Despite the huge differences in thread length and insertion depth of the two screw types, insertion times did not show significant differences within the same rotational speed. This is likely due to the different thread pitches of 1.00 mm (monocortical) and 1.25 mm (bicortical) and the different self-cutting behavior of the conical pin design with its varying screw diameter.

In conclusion, our findings suggest that during insertion of self-drilling bicortical external fixator pins with power drills, the resulting bone debris create temperatures which can be potentially harmful to the surrounding tissue. This risk is significantly reduced using unicortical pins in particular with low rotational speeds. The temperature increase within the bone tissue similarly is much lower with unicortical pin insertion compared with bicortical pins. Thus, our findings suggest that the in vivo application of unicortical, self-drilling fixator pins will not be associated with risk for thermal damage, neither to the surrounding tissue nor to the bone itself.
